# The Multifaceted Role of the Vasculature in Endochondral Fracture Repair

**DOI:** 10.3389/fendo.2015.00004

**Published:** 2015-02-05

**Authors:** Chelsea S. Bahney, Diane P. Hu, Theodore Miclau, Ralph S. Marcucio

**Affiliations:** ^1^Orthopaedic Trauma Institute, San Francisco General Hospital, University of California San Francisco, San Francisco, CA, USA; ^2^Department of Bioengineering and Material Science, University of California Berkeley, Berkeley, CA, USA

**Keywords:** fracture repair, endochondral ossification, cartilage transformation, angiogenesis, bone biology

## Abstract

Fracture healing is critically dependent upon an adequate vascular supply. The normal rate for fracture delayed or non-union is estimated to be between 10 and 15%, and annual fracture numbers are approximately 15 million cases per year. However, when there is decreased vascular perfusion to the fracture, incidence of impaired healing rises dramatically to 46%. Reduction in the blood supply to the fracture can be the result of traumatic injuries that physically disrupt the vasculature and damage supportive soft tissue, the result of anatomical location (i.e., distal tibia), or attributed to physiological conditions such as age, diabetes, or smoking. The role of the vasculature during repair is multifaceted and changes during the course of healing. In this article, we review recent insights into the role of the vasculature during fracture repair. Taken together these data highlight the need for an updated model for endochondral repair to facilitate improved therapeutic approaches to promote bone healing.

## Introduction

Bones are highly vascularized tissues with a diverse and central role in normal physiology; normal functions include, regulating systemic homeostasis, enabling bone turnover, and providing a niche for hematopoiesis. As such, it is necessary for bone regeneration to reestablish both the embedded osseous vasculature and the bone marrow cavity in order to restore full functionality to the tissue. Delay or compromise to the angiogenic process during fracture repair has a significant effect on the progression of proper bone healing. Ischemia at the fracture site dramatically reduces the rate of fracture repair and is a major risk factor for non-healing fractures, which are clinically termed “non-union” (1, 2). With an estimated 15 million fractures a year, and a delayed- or non-union rate of close to 50% when fracture occurs in conjunction with severe vascular injury, treatment of recalcitrant fractures represents a significant burden on the healthcare system (3, 4).

Fracture non-union is a clinical diagnosis made when a patient has clinical symptoms that include pain, instability, and poor radiographic bone healing at a time point after which healing should be expected. Classically, three major types of non-unions have been described – hypertrophic, atrophic, and oligotrophic – and are diagnosed based on the radiological evidence of bone formation. Atrophic non-unions are classically associated with a poor biological response, including an insufficient blood supply (5), and a major challenge in treating these cases is the restoration of the vasculature or stimulation of new blood vessel formation. In contrast, hypertrophic non-unions are associated with an adequate biological response in the presence of excessive instability at the fracture site, and in some cases can be successfully treated by improved stabilization of the fracture. Oligotrophic non-unions are not hypertrophic, but characteristically are vascular with no callus formation due to a severely displaced fracture. The treatment of non-unions frequently results in long-term patient disability, loss of wages, and can require multiple surgeries to achieve union.

Bone grafting is one technology used to stimulate bone regeneration in cases of fracture non-union, or for a number of other clinical indications that require bone formation be augmented (i.e., spinal fusions, post-traumatic gap defects, tumor resections, craniofacial reconstruction, and complex joint replacements) (6). Autograft bone is the gold standard treatment in these cases and generally produces good clinical outcomes. The most common form for autografts is morselized cancellous bone from the iliac crest, which effectively remodels and includes angiogenic factors from the bone marrow in order to promote regeneration of a vascularized bone. Alternatively, structural vascular autografts can be utilized for repair. Recently procedures have been developed to include the vascularized pedicle in the transplant, which offers a great potential to improve outcomes, but the procedure is technically very challenging and often requires additional surgical subspecialties become involved. Regardless of the form factor of these grafts, the clinical utility of autograft bone is significantly limited by the availability of tissue for transplantation and resultant donor site morbidity.

Devitalized bone allografts have been developed to address some of the clinical shortcomings of autografts. Unfortunately, failure rates associated with allograft procedures are estimated between 16 and 35% (7). The underlying cause for autograft failure is typically associated with their limited ability to remodel: poor cellular and vascular invasion into the graft, along with poor osseointegration between the graft and host tissues, results in loss of mechanical strength and graft displacement.

The necessity of the bone regenerate to revascularize for proper function is the end-result of a cascade of carefully coordinated events that require the vasculature. This review provides an overview of how the vasculature contributes throughout the process of fracture healing to create a hematoma, deliver cells, modulate oxygen tension, and deliver growth factors involved in the healing process. Here, we focus only on the process of indirect bone healing, where bone is formed following a cartilage intermediate in a process called endochondral ossification. The evidence presented from this compilation of data illustrates an explicit need to update the existing model of endochondral bone repair, and emphasizes the potential opportunity for novel therapeutic treatments for bone repair that focus on the chondrogenic and angiogenic stages of repair.

## Hematoma and Immune Response Stimulate Repair

The essential role of the vasculature in bone healing becomes evident at the onset of the injury as a fracture typically exposes the bone marrow cavity and disrupts blood vessels within the bone and surrounding soft tissues. Formation of a hematoma around the trauma is a critical first step in fracture healing and its absence will delay healing (8). The hematoma contains the fracture debris and initiates a pro-inflammatory cascade by recruiting immune cells from the surrounding soft tissues, lymphatic system, and vasculature. Within the hematoma a low pH microenvironment is formed where inflammatory cells secrete pro-inflammatory cytokines [tumor necrosis factor α (TNFα), interleukin-1β (IL-1β), and IL-6] to activate the polymorphonuclear neutrophils and M1 (or pro-inflammatory) macrophages that are recruited to manage this acute inflammation stage (9). The fibrin network formed by the hematoma serves as a temporary scaffold for these leukocytes and this early pro-inflammatory phase has a demonstrated positive effect on fracture healing (10). Specifically, the pro-inflammatory phase promotes cell proliferation [IL-1β (11) and basic fibroblast growth factor, FGF2 (12)], cell differentiation [matrix metalloproteinase-9, MMP9 (13), and bone morphogenetic protein, BMP (14)]. Lactate levels are also high during the pro-inflammatory stage (15–19), which leads to an upregulation of angiogenic factors such as angiopoetin-1, platelet derived growth factor (PDGF), and vascular endothelial growth factor (VEGF) (20, 21). For additional details see reviews (22–25).

**Figure 1:**
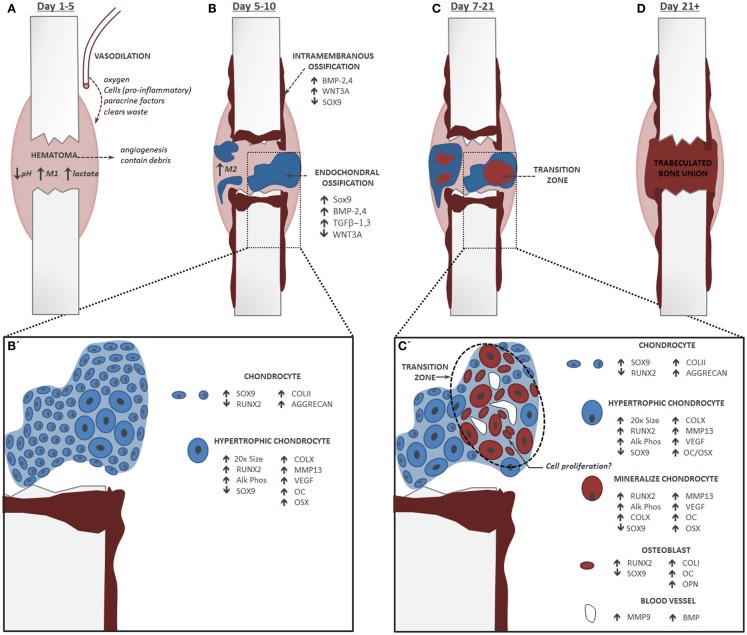
**Endochondral Fracture Schematic: A fracture that is not rigidly stabilized will heal through a combination of intramembranous and endochondral ossification**. Fracture healing progression: bone is depicted in red and cartilage in blue, time course corresponds to healing in a mouse tibia. **(A)** The first stage of fracture healing is formation of a hematoma to initiate an inflammatory cascade characterized in part by an abundance of pro-inflammatory M1 macrophages, low pH, and elevated lactate level. **(B)** Next, local osteochondral progenitors differentiate into bone and cartilage to initiate healing. Intramembranous ossification occurs primarily at the periosteum and endosteum of the fracture callus, while the soft cartilage callus forms in the central portion of the fracture where there is maximal mobility. For healing to progress normally, inflammation must be down-regulated and there is a shift in the macrophage population from a pro-inflammatory M1 state to an anti-inflammatory M2 state. **(B’)** Within the cartilage callus chondrocytes differentiate and mature in a parallel fashion to the growth plate. **(C)** Conversion of cartilage to bone during endochondral ossification occurs concomitantly with the invasion of blood vessels. **(C’)** Blood vessels lead to mineralization of the cartilage matrix and new data suggest that these cells transform directly into osteoblasts. Chondrocytes do not undergo significant apoptosis and may re-enter the cell cycle. **(D)** The cartilage callus becomes fully converted to a trabeculated bone that will bridge the full fracture defect. This trabeculated structure will be remodeled into a cortical bone that is almost indistinguishable in form and function from the native bone.

While activation of the fracture repair process seems dependent on an adequate pro-inflammatory response, resolution of this inflammatory state is critical to progression of healing. The anti-inflammatory state is the due largely to phenotypic modulation of the macrophages to the M2 (or alternatively activated, anti-inflammatory macrophages) population in the fracture callus. These anti-inflammatory macrophages secrete a battery of cytokines and growth factors to promote tissue repair and angiogenesis, such as, IL-10, PDGF, VEGF, transforming growth factor-β (TGFβ), epidermal growth factor (EGF), and arginase (26). During fracture a sustained pro-inflammatory state due to dysregulated macrophage polarization appears to be an underlying mechanism for delayed healing in conditions such as older age and disease (27–29) (Figures 1A,B).

## Determinants of the Fracture Healing Pathway

Following the hematoma and early inflammation stage, fracture healing can proceed through two different pathways: intramembranous or endochondral ossification. Intramembranous ossification is the process of direct bone formation, where osteochondral progenitors differentiate directly into osteoblasts. Osteogenesis is mediated by the co-expression of the runt-related transcription factor 2 (RUNX2) (30–32) and β-catenin (33), which is the critical transcriptional co-factor for canonical WNT signaling. Together, activation of these pathways, along with a morphological shift from the fibroblastic appearance of the progenitor cell into a more columnar appearance, phenotypically defines the osteoblast. Osteoblasts synthesize an extracellular matrix containing Type I collagen and coordinate matrix mineralization in a highly regulated process that is not fully understood (34–36). During intramembranous healing, bone spicules created from the osteoblasts fuse to form a trabeculated bone that is eventually fully remodeled into a cortical bone ultrastructure through the coordinated action of osteoblasts and osteoclasts.

Endochondral ossification is the process of indirect bone formation in which bone is formed through a cartilage intermediate. The cartilage portion of the fracture callus results from chondrogenic differentiation of periosteal progenitor cells. Chondrogenesis is initiated by the expression of SOX9, a member of the SRY-related high-mobility group box transcription factor family required for chondrogenesis (37). SOX9 regulates downstream synthesis of Type II collagen and aggrecan (38–41), which are the canonical markers of cartilage. Chondrogenic and osteogenic programs in the progenitor cell pool oppose each other at a molecular level. For example, activation of WNT/β-catenin during osteogenesis specifically prevents differentiation toward the chondrogenic lineage by suppressing SOX9 (42, 43).

During fracture healing, both intramembranous and endochondral ossification typically contribute in part to the healing process, but dominance of healing by one pathway versus the other is influenced largely by stability at the fracture site. Reduction, or realignment, of the fracture is a primary goal in successfully treating the bone to promote healing. Full reduction, which results in complete stability through the application of fixators such as bone plates, will promote healing by intramembranous ossification. More commonly, fractures are reduced to enable some motion, such as those treated with an intramedullary nail. This promotes a more robust healing reaction in which endochondral ossification is the primary healing response within the fracture gap (Figure 1B).

In addition to the mechanical stability of the fracture, the origin of the progenitor cells that contribute to the fracture callus also influences the bone healing pathway by providing cells with distinct differentiation potentials. The osteochondral progenitors that give rise to the fracture callus are recruited locally from the endosteum and periosteum of the fractured bone (44). The cells from the endosteum preferentially undergo intramembranous ossification to directly form osteoblasts and stimulate bridging bone within the marrow cavity during repair. Conversely, the periosteal cells appear to be more bi-potent with their fate dictated by mircoenvironmental forces (45). In fractures that are not rigidly fixed, these cells will undergo both osteogenesis and chondrogenesis in a location specific manner. Along the periosteal surface of the bone, where there is no motion, cells undergo intramembranous ossification; whereas in the fracture gap, the progenitor cells undergo chondrogenesis to form bone through endochondral ossification (Figure 1B). While it has been noted that circulating progenitor cells are recruited to the fracture site during bone regeneration, data suggests that their contribution directly to the fracture callus is minimal (44, 46, 47).

## Endochondral Fracture Repair

The molecular and cellular mechanisms that control endochondral ossification are more complex than intramembranous ossification. In this pathway, SOX9 expression promotes condensation and commitment of the osteochondral progenitors toward the chondrogenic lineage. After specification, subsequent SOX9 activity is necessary to maintain cell morphology and the chondrogenic phenotype through maturation to hypertrophy. In a process that resembles the well-described process of endochondral ossification in the growth plate (48), cartilage within the fracture callus contains a pool of proliferating chondrocytes that successively undergo hypertrophic maturation prior to attaining a bone phenotype (Figures 1B,C). While the fracture callus lacks the concise organization of the growth plate, SOX9 expression is easily detected in the early fracture callus condensation demonstrating nucleation of chondrogenesis. Subsequently, SOX9 regulates production of the cartilage extracellular matrix by directly binding to the enhancer elements that control collagen II and aggrecan expression (38–40, 49).

Similar to growth plate chondrogenesis, chondrocytes within the fracture callus also undergo a controlled and progressive matruation. Hypertrophic maturation of the chondrocyte is marked by a strong expression of collagen X in the area where cartilage transitions to bone. These hypertrophic chondrocytes are highly bioactive to promote mineralization and vascular invasion of the cartilage matrix through potent expression of BMP (50), MMP-13 (51), alkaline phosphatase, VEGF (52–54), and PIGF (placental growth factor) (55).

**Figure 2:**
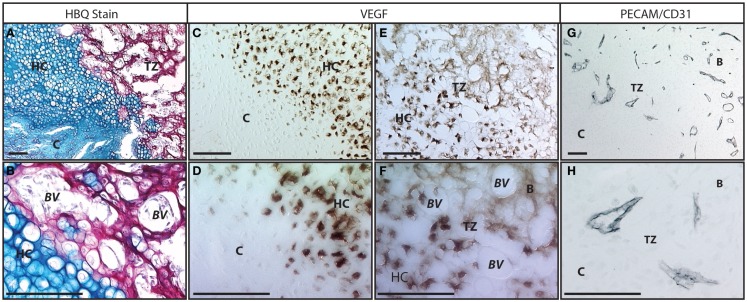
**Vasculature in the fracture callus transition zone: (A,B) Histology of fracture callus 10 days following injury stained with Hall’s and Brudt’s Quadruple (HBQ) stain that indicates the cartilage matrix in blue through alcian blue and the bone matrix in red through direct red dye**. We have defined the region where the cartilage becomes bone in the fracture callus as the transition zone (“TZ”). In this region, the cartilage (“C”) matures through hypertrophy (“HC”) and junctions into bone in an area that is clearly marked by the blue to red matrix transition and the invasion of blood vessels (“BV”). Within the red bone matrix (“B”) cells with the large round hypertrophic chondrocyte morphology and the smaller elongated osteoblast morphology can be clearly identified. **(C–F)** Immunohistochemistry to VEGF protein. **(C,D)** The transition from normal to hypertrophic cartilage demonstrates that only hypertrophic chondrocytes make VEGF. **(E,F)** At the hypertrophic cartilage to bone transition this VEGF is responsible for recruiting the vasculature. Osteocytes no longer make VEGF protein but the protein binds to the extracellular matrix in this transition zone. **(G,H)** Immunohistochemistry to PECAM/CD31 protein marks the vasculature invading in the transition zone. Scale bar = 100 μm; B = bone, BV = blood vessel, C = cartilage, HC = hypertrophic cartilage, TZ = transition zone.

The conversion of cartilage to bone in the fracture callus occurs adjacent to the invading vasculature in a domain that we will refer to as the “transition zone.” The cartilage in this transition zone becomes calcified and the extracellular matrix stains as bone by classic Trichrome or Direct Red histology techniques, yet the cells retains their hypertrophic morphology (Figures 2A,B). These hypertrophic chondrocytes also demonstrate significantly overlapping phenotypes with other distinct bone markers, such as collagen I, osterix (OSX), osteocalcin (OC), and osteopontin (OPN) (56–59). The ultimate fate of the hypertrophic chondrocytes prior to bone formation has been debated and will be discussed separately below. However, the general steps that are agreed upon are: that the calcified cartilage matrix is resorbed, replaced by bone, and remodeled into functional bone that resembles the original cortical bone through the action of osteoblasts and osteoclasts/chondroclasts.

## Role of Oxygen in Fracture Healing

Oxygen tension in the fracture callus changes during the process of endochondral bone formation (27), having both a direct and indirect effect on healing. As is the case for any tissue, oxygen delivery by the vasculature has a direct effect on cell survival. Ischemic fractures created in a murine model demonstrate increased apoptosis of the periosteal progenitor cells to produce delayed fracture healing in a manner that parallels the clinical condition (1, 2). Decreased oxygen at the fracture (27) may contribute to the production of angiogenic factors (20) and vasodilation (9) of the soft tissues surrounding the fracture. Stabilization of hypoxia-inducible factor 1α (HI-1α), directly regulates *Sox9* expression in the condensed mesenchymal cells to promote chondrogenesis, enable chondrocyte survival, and enhances extracellular matrix synthesis (60–62). Interestingly, systemic changes in oxygen tension do not significantly alter chondrogenesis during the early stages of fracture healing, but hyperoxia increases tissue vascularization and rescues delayed healing in ischemic fractures (21). Further, increasing angiogenesis by removing anti-angiogenic signals from thrombospondin-2 stimulates healing of ischemic fractures (63).

## Angiognesis in the Fracture Callus

During endochondral repair, the fracture callus remains avascular during the initial soft callus phase. However, as chondrocytes within the callus mature to hypertrophy, they become potent stimulators of angiogenesis and vascular invasion by secreting VEGF (52–54), PIGF (55), and PDGF (64) (Figures 2C–H). The importance of angiogenesis to the progression of fracture healing has been experimentally demonstrated by inhibiting VEGF through delivery of a soluble neutralizing VEGF receptor (Flt-IgG) to produce delayed conversion of the cartilage callus to bone following impaired vascular invasion (53, 65). These results are supported by similar studies where animals receiving the anti-angiogenic immunosuppressant Rapamycin demonstrated significant delays in endochondral repair (66).

Further evidence for the importance of angiogenesis in fracture repair is found in the clinical studies demonstrating delayed fracture healing as a result of smoking. Compared with an estimated 9% rate of open-tibia non-union in the non-smoking population, the LEAP study found smokers presented with a 24% chance of non-union and that the fractures are more recalcitrant to further intervention to stimulate healing. A study by Ueng et al. suggests that one underlying mechanism for this delay is the diminished vascularization induced by smoking (67). While many studies have hypothesized that smoking disrupts angiogenesis directly, it has not been proven.

In addition to delivering oxygen and enabling gas exchange, new blood vessels also deliver general nutrients necessary for cell survival and provide an egress for waste products. Blood vessels supply a number of circulating factors that are important to normal fracture healing, such as, parathyroid hormone (PTH), insulin, and Vitamin D. Importantly, vascular invasion also corresponds with calcification of the cartilage matrix and transition to bone. The precise molecular mechanisms, and location of signaling, which facilitate mineralization of the cartilage at the fracture callus is not clear. Changes in calcium concentration are sufficient to induce mineralization of these hypertropic chondrocytes, yet it remains unclear what the source of calcium is and which cells sense these changes. Mineralization of the cartilage matrix is also initiated by osteoinductive signals, such as BMP, secreted by both the chondrocytes themselves (50), and by the vascular endothelial cells (68, 69).

Conversion of calcified cartilage to bone requires that the cartilage matrix is degraded and replaced by bone matrix. Major differences in the extracellular matrix composition include a conversion of collagen II in cartilage, to collagen I in bone, and degradation of the glycosaminoglycans (GAGs) in cartilage. It remains debated how the extracellular matrix is remodeled during this conversion. Hypertrophic chondrocytes make MMP-13, which is one of the major enzymes responsible for degrading both collagen II and aggrecan, the major GAG in cartilage. Furthermore, the vascular endothelial cells secrete MMP-9, one of the gelatinases with a high specificity for degraded collagens, thereby accelerating cartilage degradation upon vascular invasion. Alternatively, a cellular degradation of the cartilage matrix may be occurring through the action of osteoclasts that are delivered to the cartilage matrix through the vasculature. Osteoclasts are recruited to calcified cartilage both by production of the receptor activator of NF-κB ligand (RANKL) (70, 71) in the hypertrophic cartilage, and by MMP-9 expression in the vasculature (13). Some argue the cellular contribution of the osteoclasts is not required for fracture remodeling (72), while others claim there is a specialized osteoclasts, called the “chondroclast” (73, 74), which is unique to cartilage degradation versus bone. In addition to converting the cartilage matrix to bone matrix, this remodeling phase also requires a large portion of the solid matrix be removed to create marrow space within the highly trabeculated bone structure that is initially formed during boney remodeling (Figure 2A).

## Ultimate Fate of Fracture Callus (Hypertrophic) Chondrocytes

The commitment and progression of the chondrocyte during endochondral ossification is a long-standing question that is not fully understood. In the proliferating and pre-hypertrophic chondrocytes, SOX9 [and associated co-factors SOX5/6 (38)] establishes chondrocyte identity and actively represses RUNX2 expression. In this state, hypertrophic maturation is actively inhibited through a complex negative feedback regulatory mechanism in which Indian Hedghog (IHH) activates PTHrP (parathyroid hormone related protein) through the molecular mediators Gli and Patched [see reviews (48, 75–77)]. Hypertrophy proceeds when loss of PTHrP relieves repression of chondrocyte maturation. Morphologically, the hypertrophic chondrocyte is identified by a dramatic increase in cell size. A recent study quantified this growth as a ~20-fold increase in both volume and dry mass (78). The molecular mechanisms that regulate this enlargement are unclear, but are likely modulated by sodium ion channels (79) and can be induced by thyroxine (80), BMPs (81, 82), and insulin-like growth factor (IGF) (83). The phenotype of the hypertrophic chondrocyte is also unique relative to earlier maturation states; a loss of SOX9 is accompanied by expression of collagen X (84), the canonical marker of the hypertrophic chondrocyte, along with upregulation of markers traditionally related to a bone phenotype such as RUNX2, alkaline phosphatase, collagen I, osterix, osteocalcin, and osteopontin (56–59).

### Terminal fate of the hypertrophic chondrocyte

Traditionally, it was believed that the hypertrophic chondrocytes exits the cell cycle (85) and represent the terminal differentiation state. This long held view is standard in the literature referring to both growth plate biology (48, 86) and fracture repair (25, 87). According to this model, hypertrophic chondrocytes undergo programed cell death (86, 88–90), then osteoblasts or osteochondral progenitor cells are delivered via the vasculature to form the bone matrix that replaces the cartilage (91). Thus a direct role for the vasculature in endochondral bone formation was thought to involve delivery of precursor cells (Figure 4).

### Transformation of hypertrophic chondrocytes to osteoblasts and osteocytes

**Figure 3:**
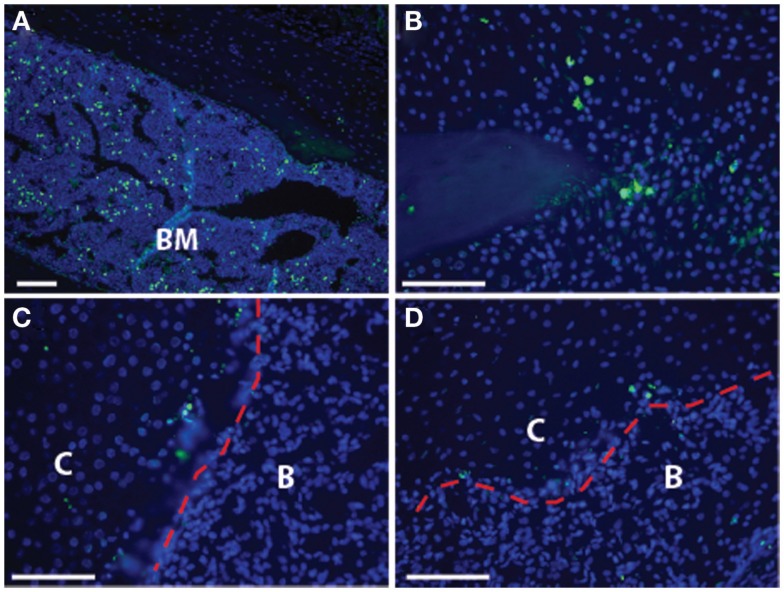
**Cell death is not pervasive at the transition zone: the established model of endochondral ossification includes cell death of the hypertrophic chondrocytes**. Here, we show TUNEL staining in the transition zone of the fracture callus to demonstrate that there is minimal cell death seen in these hypertrophic chondrocytes. **(A)** Bone marrow, as a positive control; **(B)** non-hypertrophic cartilage at tip of fractured bone; **(C,D)** transition zone with red dotted line indicating the demarcation between cartilage (“C”) and bone (“B”) that can be easily distinguished by cell morphology and bright field microscopy. Scale bar = 100 μm.

**Figure 4:**
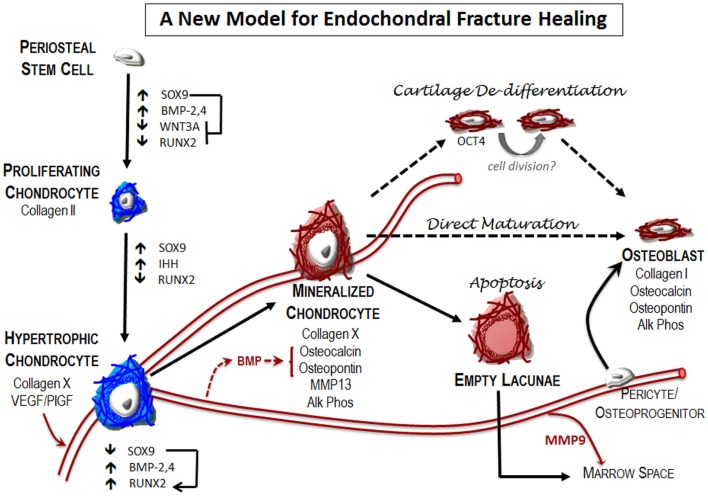
**A new model for endochondral fracture repair: local osteochondral progenitors from the periosteum and endosteum are the stem cells that differentiate to form bone and cartilage in the fracture site**. To generate the cartilage callus, osteochondral progenitors differentiate into chondrocytes (blue) that proliferate to generate the early soft callus. Chondrocytes within the callus mature into hypertrophic chondrocytes. Expression of angiogenic factors by these cells results in vascular invasion into the previously avascular soft callus. Mineralization of the hypertrophic cartilage occurs at this transition zone where blood vessels are invading. Hypertrophic chondrocytes begin to express many of the canonical markers of the osteoblast (red), including osteocalcin, osteopontin, and alkaline phosphatase. The fate of these mineralized hypertrophic chondrocytes remains unclear. Apoptosis is the classical fate ascribed to these cells. According to this model, new bone is formed by osteoblasts that arise from osteoprogenitor cells brought in through the invading vasculature. In addition, new reports indicate that at least a part of the newly formed bone in the fracture callus is chondrocyte derived. The mechanisms that enable this phenotypic conversion of cartilage to bone remain unclear. Here, we depict two proposed pathways – chondrocyte de-differentiation and direct maturation – that have been suggested in the literature, but without fully substantiated details as suggested by dotted lines.

In contrast to the model described above, an alternative mechanism for bone formation directly from the chondrocyte has been reported. Recently, we published a paper demonstrating that cartilage can transform into bone during endochondral bone repair using a number of different lineage tracing strategies (68). This work has now been followed by two complementary studies that demonstrate hypertrophic chondrocytes become osteoblasts and osteocytes during endochondral bone formation by following the fate of cells that expressed the collagen X or aggrecan gene during murine long bone development and fracture healing (92, 93). These recent murine studies give credence to earlier studies from zebrafish (94), avian (95–97), and rabbit (98) models, which reported chondrocytes were capable of forming bone directly. Together with other studies that have focused on cell death in the hypertrophic chondrocytes (99–102), these data suggest that the hypertrophic chondrocyte may not necessarily be destined for apoptosis. By focusing on the transition zone in murine fractures, we have found very little evidence of cell apoptosis using TUNEL staining (68) (Figure 3). While these data do not exclude the possibility that a subset of the hypertrophic chondrocytes undergo apoptosis, a process that is necessary to create physical space for the bone marrow cavity, it does indicate that this is not the absolute fate of the hypertrophic chondrocyte (Figure 4).

The mechanism by which chondrocytes transform into osteoblasts remains unclear. One possible mechanism is that chondrocytes mature directly into osteoblasts, and that the expression of canonical bone markers in the hypertrophic chondrocyte represents the natural phenotypic progression of endochondral chondrocytes (95, 98, 103–108). The phenotypic plasticity of these endochondral chondrocytes was highlighted in a study showing that loss of SOX9 expression in pre-hypertrophic chondrocytes results in the conversion to osteoblast identity (89).

Another possibility is that the chondrocyte can “de-differentiate” into a progenitor-like state before becoming bone (68, 109, 110). Supporting this mechanism, we recently showed that hypertrophic chondrocytes at the transition zone of the fracture callus express the progenitor marker Oct4A (68). These possibilities are not mutually exclusive; epigenetic remodeling in the hypertrophic chondrocyte by histone deacetylases activated by the pluripotent stem cell programs may enable chromatin remodeling to expose alternatively suppressed bone genes. Furthermore, it has been suggested that the endochondrally derived osteoblast may arise from an asymmetric cell division of the hypertrophic chondrocyte (101, 102). Hypertrophic chondrocytes are traditionally considered to be a post-mitotic cell type: activation of the progenitor cell genes may be a critical step in allowing these cells to re-enter the cell cycle.

The transition zone is always immediately adjacent to the invading vasculature (Figures 1 and 2). Given that the vasculature expresses osteogenic molecules (69), one exciting role for the vasculature may be in stimulating the progression of the hypertrophic chondrocyte to become an osteocyte. While, further work is required to demonstrate this conclusively, our previously published data show that conditioned media from endothelial cells causes the cartilage matrix derived from a fracture callus to mineralize, the first step in the conversion of cartilage to bone (68).

## Concluding Remarks: A New Model for Endochondral Repair

Improving our knowledge of the basic mechanisms underlying normal and delayed fracture repair are central to developing improved therapies for bone healing. The information presented here suggests that the traditional view of endochondral bone repair needs to be updated in light of new data (Figure 4). Specifically, these data suggest that the vasculature has a critical role in all phases of fracture healing and that there are multiple potential targets for therapeutic intervention. Understanding how the immune response regulates fracture repair is perhaps the first opportunity to positively influence healing outcomes. The hematoma formed initiates the inflammatory and angiogenic responses that are required for appropriate healing.

During endochondral bone repair, formation of a soft cartilaginous callus is the next milestone in fracture healing. The cartilage itself is a highly angiogenic tissue that may be the primary mechanism for recruiting and establishing a functional vasculature and marrow space in the new bone. In turn, the invading vasculature is central in coordinating the conversion of the cartilage matrix to bone, both by providing osteoinductive signals (e.g., BMP, others), and by bringing osteoclasts in to facilitate remodeling of the tissue. Classically, it is also believed that the vasculature was the source of the osteoblasts that form the new bone. Newer data suggest that chondrocytes themselves directly contribute to the new bone. While this finding does not exclude the possibility that cells from the invading vasculature do not contribute to endochondral bone formation (91), it does suggest that invading cells may not be the only, or even primary, source of cells that form bone during endochondral repair (68, 92, 93, 98, 107).

After the cartilage is converted to bone the tissue is highly trabeculated. Remodeling is the final phase of healing: it allows for complete bridging of the fracture site, structural integrity, and the maintenance of the marrow cavity for hematopoiesis. This process ultimately produces a cortical bone structure that is nearly identical to the original bone in both form and function through a tightly regulated coupling of osteoblast and osteoclast activity.

## Conflict of Interest Statement

The authors declare that the research was conducted in the absence of any commercial or financial relationships that could be construed as a potential conflict of interest.

## Abbreviations

BMP, bone morphogenetic protein; EGF, epidermal growth factor; FGF, fibroblast growth factor; GAG, glycosaminoglycans; HIF, hypoxia inducible factor; IGF, insulin growth factor; IHH, Indian hedgehog; ILβ, interleukinβ; M1, pro-inflammatory macrophage; M2, anti-inflammatory macrophage; MMP, matrix metalloproteinase; OC, osteocalcin (aka bone gamma-carboxyglutamic acid-containing protein, BGLAP); OCT4, octamer-binding transcription factor 4; OPN, osteopontin; OSX, osterix (aka SP7); PDGF, platelet derived growth factor; PECAM, platelet endothelial cell adhesion molecule (aka CD31); PIGF, placental growth factor; PTH, parathyroid hormone; PTHrP, parathyroid hormone related protein; RANKL, receptor activator of nuclear factor kappa-B ligand; RUNX2, runt-related transcription factor 2 (aka CBF-α1); TGFβ, transforming growth factor β; TNFα, tumor necrosis factor alpha; VEGF, vascular endothelial growth factor.
